# Book Review

**Published:** 1908-03

**Authors:** 


**The Medical Annual, 1907.** Bristol: J. Wright & Son.—Once more we have to welcome this almost indispensable annual. It is too well known to require anything like an introduction to our readers, and in saying that it represents an even further advance on the merits of its predecessor we bestow on it the highest encomium. We are glad to see that the stereoscopic plates are as good and useful as those in the earlier volumes. We strongly advise those who are not already subscribers to become so.

**Clinical Lectures on Neurasthenia.** By Thomas D. Savill, M.D. Third Edition. Pp. xvi., 216. London: H. J. Glaisher. 1906.—The fact that this book has reached a third edition in seven years indicates that it it has been found useful by a number of readers. The present edition profits by some additions and excisions, which the author's experience has shown him to be advisable, but follows the same general plan as the previous ones. It retains the form of lectures; not, in our opinion, the most satisfactory one for a book of this length on a single disease. The author maintains his position as to the autotoxic origin of neurasthenia from gastro-intestinal disorder; this, however, rests on no more certain evidence than the frequency and antecedent occurrence of gastro-intestinal derangements in neurasthenia. At the same time, the author does not deny that in a number of instances causes roughly classified under the term "mental" play an important part. In the section on cerebral symptoms he seems to be trying, under the name of "neurasthenic insanity" to add another variety to the already involved nomenclature of insanity. We do not think that he has made out his case, although the characteristic of bodily weakness or other physical disorder preceding and accompanying the mental disorder is a distinction of some importance, it is hardly sufficiently so to be diagnostic of a variety. Nor does his proposed classification of insanity seem to us particularly illuminating, nor one by which real advance is likely to be made. At the same time the chapter is an interesting one. The section on treatment will be found useful, and the book is eminently readable. It concludes with a bibliography, which has been brought up to date.

**Transactions of the American Dermatological Association.** Thirtieth Annual Meeting, 1906. Report of the Proceedings by GROVER W. WENDE, M.D.—As usual, these *Transactions* comprise a useful and interesting series of excellently-reported dermatological cases, with beautifully reproduced photographs of the lesions, macro-and microscopic.

Among the papers may be mentioned particularly one from Robert W. Taylor, who advances the view that in syphilis the virus is spread rapidly by means of the veins and not lymphatics, the chancre being an expression of the earliest local effect.

Also a paper by Engman and Mook, in which they show that lesions of the skin are produced by means of the blood vessels, and not the skin glands in bromide and iodide eruptions. There is a useful report on a case of pemphigus vegetans by J. M. Winfield, with an excellent review of the reported cases. The finding of the B. pyocyaneus in pure culture in the blood, and the same bacillus and staphylococcus aureus in the lesions, is of importance.

Breakey's paper on "phagedenic and serpiginous ulcers and infective granulomata" is also of importance.

The usual summary of the dermatological cases of the year is always of value, and might be imitated in England with advantage.

**Department of Neurology.** Harvard Medical School. Vols. I. and II. Boston, Mass. 1906—1907.—These volumes consist of papers on neurological subjects contributed by members of the staffs of the Massachusetts General Hospital, the Boston City Hospital, the Long Island Hospital and the Neurological Laboratory to various medical journals during each year respectively, and collected in this way for future reference. The collected papers are worth perusing, and reflect credit on the work of the hospitals concerned. They will repay perusal, not only by the neurologist, but by the general practitioner.

**The Quarterly Journal of Medicine.** Vol. I., No. 1. Oxford: The Clarendon Press. October, 1907.—This is the first number of a new journal which has been founded by The Association of Physicians of Great Britain and Ireland, by whom the editorial committee and a body of collaborators were appointed at the first annual meeting (1906). One hundred and fifty-five members attended the meeting. The papers now published are thirteen, of the highest scientific value, and such as would be expected from the distinguished editors whose names appear on the title-page.

**Post-Graduate Clinical Studies for the General Practitioner.** By H. HAROLD SCOTT, M.B. Pp. ix., 166. London: H. K. Lewis. 1907.—This volume contains a collection of nine instructive essays which for the most part have been read by the author at medical meetings. The object of the writer, who has certainly succeeded in his attempt, has been to bring forward questions, mainly connected with diagnosis, which are of special interest to the general practitioner, and to discuss them at much greater length than is possible in the ordinary text-books. The author has treated his subjects throughout in a thoroughly practical manner, and quotes very largely from his own experience when discussing the various problems. The last chapter differing from the rest in scope, deals with the question of syphilis in the lung.

**Bilharziosis.** By FRANK COLE MADDEN, M.D. Pp. 78. London: Cassell and Company Limited. 1907.—Dr. Madden's monograph—based as it is upon a long experience of bilharzial disease in Egypt—is full of instruction, especially to those of us who are not fully aware of the widespread and serious nature of the ravages of this parasite. A good account of the pathology, symptomatology and treatment is given, divided into chapters according to the systems and organs affected. We should like to know a little more of the transference of the worm from the portal to the systemic circulation, considering the importance of the urinary aspects of the disease.

The book is well printed and illustrated, and Dr. Madden has earned the thanks of the profession, at home and abroad, for giving so readable an account of his subject.

